# Gastric adenocarcinoma cutaneous metastasis arising at a previous surgical drain site: a case report

**DOI:** 10.1186/1752-1947-3-65

**Published:** 2009-02-16

**Authors:** Umberto Morelli, Roberto Cirocchi, Valerio Mecarelli, Eriberto Farinella, Francesco La Mura, Paolo Ronca, Gianmario Giustozzi, Francesco Sciannameo

**Affiliations:** 1 Università degli Studi di Perugia, Clinica Chirurgica Generale e d'Urgenza, Azienda Ospedaliera S. Maria, Terni, Italy

## Abstract

**Introduction:**

Skin metastasis from internal carcinoma rarely occurs. It has an incidence of 0.7 to 9% and it may be the first sign of an unknown malignancy. However, it can also occur during follow-up.

**Case presentation:**

A 90-year-old female patient was admitted to our surgical division with a diagnosis of anemia from a bleeding gastric adenocarcinoma. She underwent a gastric resection and Billroth II retrocolic Hofmeister/Finsterer reconstruction. She developed an enteric fistula, which needed a permanent abdominal drain until the 60^th ^postoperative day. After 12 months she was readmitted to our division with subacute small bowel obstruction and an erythematous swelling on the right side of the abdomen. Biopsies characterized it as a cutaneous metastasis from the gastric adenocarcinoma. No surgical therapy was performed given her poor clinical condition.

**Conclusion:**

Skin metastasis from carcinomas of the upper gastrointestinal tract is very rare. Persisting erythematous nodules must be biopsied in order to diagnose cutaneous metastases and to recognize them early and start prompt therapy with anti-tumour agents before the occurrence of massive visceral metastases.

## Introduction

Metastasis to the skin from internal carcinoma rarely occurs and it has an incidence of 0.7 to 9% [1,2]. Skin metastases may be the first sign of an unknown malignancy. However, it can also occur during follow-up [3,4]. The most common origin of cutaneous metastases is breast cancer in women and lung cancer in men. Skin metastases from gastric adenocarcinoma are rare [3,5]. Surgical drain site metastasis is rare, but possible [6]. It usually occurs after palliative resection in which the tumour mass remains. We describe here a patient who developed a skin metastasis from a gastric adenocarcinoma in the location of a previous surgical drain.

## Case presentation

**Figure 1:**
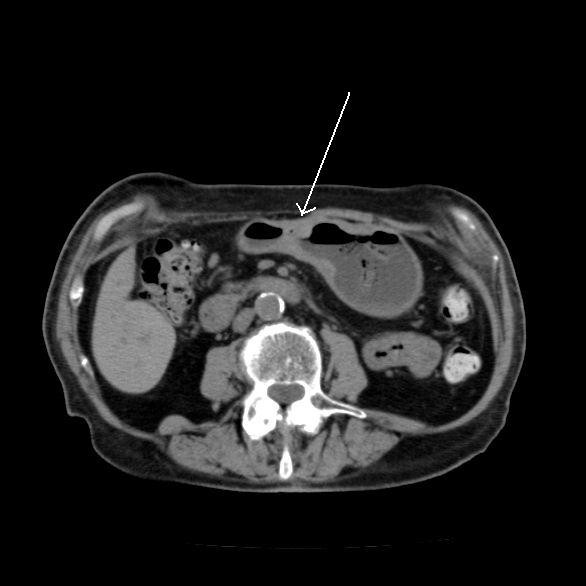
**Preoperative CT scan**. It shows the gastric adenocarcinoma of the antrum, involving both anterior and posterior gastric walls.

**Figure 2:**
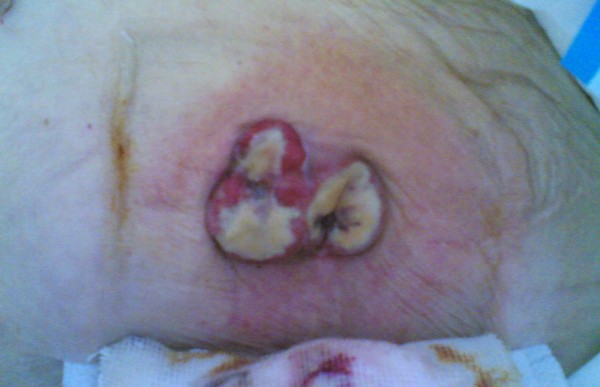
**Abdominal erythematous cutaneous swelling**. This was situated in the right abdominal wall, in the previous drain site.

A 90-year-old woman was admitted to the emergency department of our hospital because of a syncope episode. A routine blood test showed severe anaemia (Hb 6.6 gr/dl) and physical examination showed no abnormalities but traces of melaena. She had a previous history of nicotinism and ischaemic heart disease with hyperkinetic arrhythmia. She was hospitalized on a medical ward to receive blood transfusions and to undergo more diagnostic examinations. A cardiology consultation showed no abnormalities, and upper gastrointestinal endoscopy was performed. A bleeding endoluminal gastric mass was found arising from the antrum, extending to the angulus and involving both the anterior and posterior gastric wall. An adrenalin injection (1:10000) was executed on the site of the bleeding to control the haemorrhage and biopsies were collected. A surgical consultation was requested and the patient was transferred to the division of general and emergency surgery. The pathological report from the biopsies revealed a moderately differentiated gastric adenocarcinoma (G2). A computed tomography (CT) scan was also performed and confirmed the presence of a gastric mass with perigastric adenopathies (Figure 1). No evidence of metastastic activity was found. After an anaesthesiological consultation and subsequent fluid therapy to achieve a good balance, surgical intervention was scheduled. Given her age, the extent of the tumour and general clinical conditions, we decided to perform a gastric resection with D1 lymphadenectomy, and reconstruction using a Billroth II Hofmeister-Finsterer retrocolic loop with mechanical sutures. We inserted a nasogastric tube to obtain gastric decompression and a surgical drain next to the anastomosis. The pathological report disclosed a mixed type adenocarcinoma of the stomach, with signet ring cells, cellular elements typical of the Lauren's intestinal type and undifferentiated cells (pT3 N0 Mx G3 R0) with surgical rim with no evidence of pathological findings. The clinical course was normal except for haemoserous output from the abdominal drain (daily output about 300 cc). This haemoserous output transformed to enteric fluid on the 12^th ^postoperative day, with a medium output of 500 cc/day. Her clinical condition was stable and there were no indications for further surgical intervention. We put the patient on total parenteral nutrition, antimicrobial therapy and somatostatin. We medically treated the fistula and achieved a prompt clinical response from the patient with reduction of enteric fistula output. The enteric fistula developed from the duodenal stump, as showed by radiological examinations and a CT scan performed after the appearance of enteric fluid on drainage. The patient retained the abdominal drain until the 60th postoperative day when she was discharged in good health. Oncological follow-up was planned, but the patient refused any treatment. Twelve months after discharge she was readmitted to our emergency department with a diagnosis of subacute bowel obstruction and transferred to our surgical department. Physical examination defined the patient to be in poor clinical condition with an erythematous cutaneous swelling (diameter 6 cm) on the right side of her abdomen at exactly the same location as the drain was previously inserted to control the enteric fistula (Figure 2). Medical therapy with fluids and a nasogastric tube for stomach decompression was performed in order to improve her clinical condition. In addition, biopsies were collected on the erythematous lesion. The bowel obstruction resolved in 2 days with the reprise of flatus. A pathological report showed the presence of signet ring cells and neoplastic cells coming from a primary adenocarcinoma of the stomach. These cells characterized the lesion as a cutaneous metastasis from an adenocarcinoma of the stomach. Given the patient's age and her poor general condition, we decided not to remove the lesion. Her clinical condition improved in 7 days, and she was transferred to the geriatric unit of our hospital for further care.

## Discussion

Metastatic carcinoma of the skin is an uncommon occurrence, with incidence rates of 5% or less [1,2,7]. Skin metastases from gastric adenocarcinoma are very rare [3,5]. Cutaneous metastases may occur late in the course of the disease, but they can also occur at the beginning, showing a severe underlying disease. Breast cancer is one of the most common tumours to metastasize to the skin, but also lung cancer, colorectal cancer, renal cancer, ovarian cancer and bladder cancer have similar rates for cutaneous metastases of between 3.4 and 4% [8]. Also, metastases from carcinomas of the upper digestive tract have an incidence less than 1% [2]. Gastric cancer, specifically, causes only 6% of all skin metastasis. Metastasis from gastric adenocarcinoma can also have a wide distribution, presenting with wide generalized cutaneous metastases. The main feature is the histological appearance as it is similar to the primary tumour. In our case, the main component was signet ring cells with mucin and laterally displaced nuclei mixed with malignant cells; however, the undifferentiated component was almost absent. Globally, the histological features of the lesion can be similar to those of the primary tumour. Another element is the widely known possibility for tumour cells seeding in drain or trocar locations in patients operated on by laparoscopy [6,9,10]. The long permanence of abdominal drains can be an additional risk in developing cutaneous metastasis, but this incident is still rare [6-9]. Otherwise, given the poor general condition and the age of the patient, medical management of the enteric fistula was almost mandatory [11]. Because of advances in cancer therapy, patients who are diagnosed with cutaneous metastasis may live longer than before. Nevertheless, skin metastases are still a sign of poor prognosis, particularly in patients affected by lung cancer, ovarian cancer or cancers of the upper respiratory tract or upper digestive tract [2]. The prognostic value of this clinical sign is important in the management of the manifestation of the disease: the average survival is 11.4 weeks (range from 2 to 34 weeks) [12]. The treatment is palliative in most cases, although chemotherapy and radiotherapy are often used to treat these patients but in many cases the treatment presents moderate or no results. The widespread dissemination of the tumour often means an early fatality.

## Conclusion

We have described a patient with cutaneous metastasis from gastric adenocarcinoma surgically treated with no macroscopic and microscopic evidence of residual disease who developed an enteric fistula from the duodenal stump which was medically treated. The age and the immunological competence of the patient can be a fertile field for uncontrolled malignant cell proliferation. However, skin metastases from carcinomas of the upper gastrointestinal tract are very rare, especially those from gastric cancer. Persisting erythematous nodules must be biopsied in order to diagnose cutaneous metastases and early recognition of them enables prompt therapy with antitumour agents before the occurrence of massive visceral metastasis.

## Abbreviations

CT: computed tomography.

## Consent

Written informed consent was obtained from the patient for publication of this case report and accompanying images. A copy of the written consent is available for review by the Editor-in-Chief of this journal.

## Competing interests

The authors declare that they have no competing interests.

## Authors' contributions

UM conceived the study, collected data and drafted the manuscript. EF helped with bibliography and critically revised the papers. RC, VM, FLM, PR, GG and FS critically revised the papers. All authors read and approved the final version of the manuscript.
